# Plastin Family of Actin-Bundling Proteins: Its Functions in Leukocytes, Neurons, Intestines, and Cancer

**DOI:** 10.1155/2012/213492

**Published:** 2012-01-04

**Authors:** Hiroto Shinomiya

**Affiliations:** Department of Immunology and Host Defenses, Ehime University Graduate School of Medicine, Toon, Ehime 791-0295, Japan

## Abstract

Sophisticated regulation of the actin cytoskeleton by a variety of actin-binding proteins is essential for eukaryotic cells to perform their diverse functions. The plastin (also know, as fimbrin) protein family belongs to actin-bundling proteins, and the protein family is evolutionarily conserved and expressed in yeast, plant, and animal cells. Plastins are characterized by EF-hand Ca^2+^-binding domains and actin-binding domains and can cross-link actin filaments into higher-order assemblies like bundles. Three isoforms have been identified in mammals. T-plastin is expressed in cells from solid tissues, such as neurons in the brain. I-plastin expression is restricted to intestine and kidney; the isoform plays a vital role in the function of absorptive epithelia in these organs. L-plastin is expressed in hematopoietic cell lineages and in many types of cancer cells; the isoform is thus considered to be a useful biomarker for cancer.

## 1. Introduction

**Figure 1:**
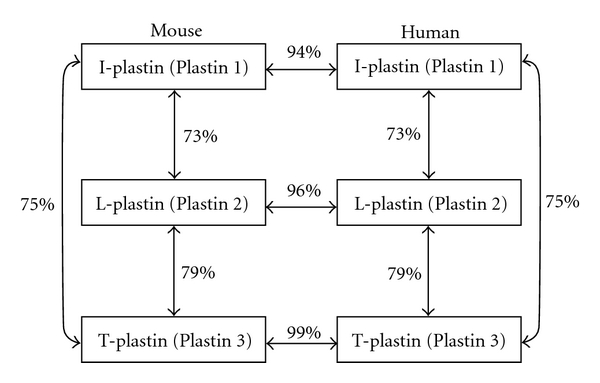
Human and mouse plastin isoforms. The homology between the amino acid sequences of these isoforms is showed.

Dynamics of the actin cytoskeleton is one of the cardinal features of eukaryotic cells, which is essential for fundamental cellular functions such as cell division, intracellular traffic of organelles, cell morphology, and cell motility [1]. The architecture of the actin cytoskeleton is regulated by a variety of proteins termed actin-binding proteins [1]. Actin filaments are organized into two types of arrays: bundles and weblike networks. Likewise, the actin filaments cross-linking proteins that help to stabilize and maintain these distinct structures are divided into two classes: bundling proteins and web-forming proteins. The plastin (also known as fimbrin) protein family belongs to bundling proteins and is evolutionarily conserved from yeast to mammalian cells. In mammals, three isoforms are known to be expressed in a cell-type-specific manner and exhibit distinct properties (Table 1 and Figure 1). In this paper, studies regarding the structure and biological functions of plastins are reviewed.

## 2. Structure of Plastins

**Figure 2:**
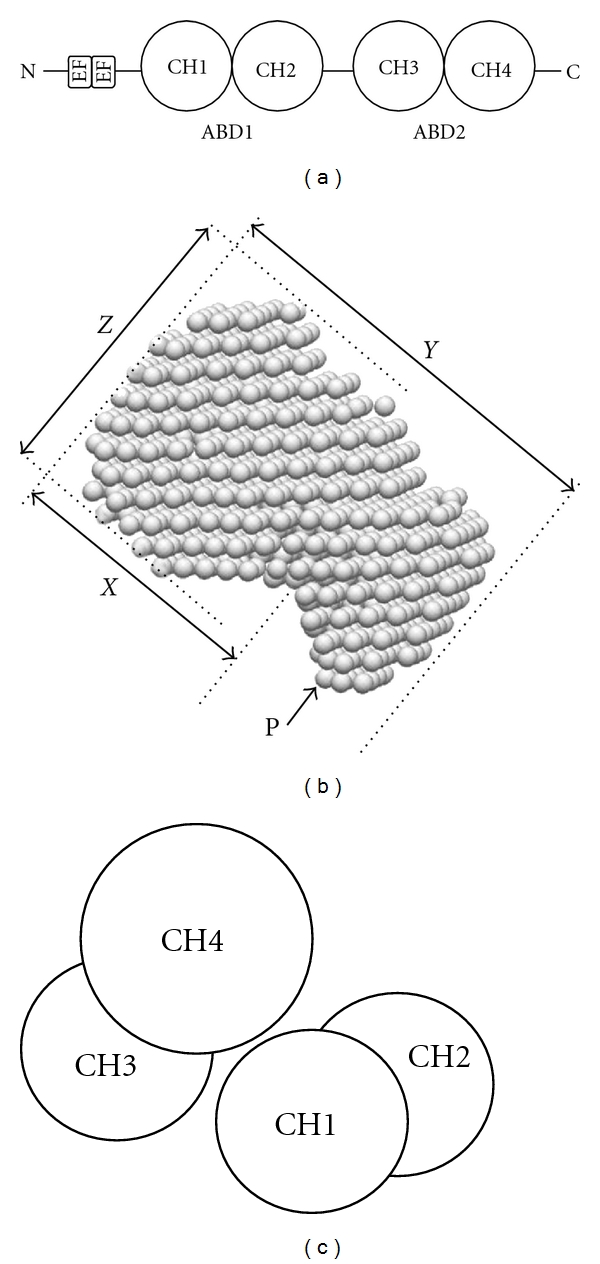
Schematic diagram of L-plastin structure. (a) Domain organization of L-plastin. The protein possesses an N-terminal headpiece of ~100 amino acids containing two EF-hand Ca^2+^-binding motifs and two actin-binding domains (ABDs) consisting of ABD1 (residues 120–379) and ABD2 (residues 394–623), and each ABD contains two calponin-homology (CH) domains. (b) Reconstructed molecular shape of L-plastin. Conformational analyses of L-plastin by X-ray scattering in solution revealed that plastin has a compact globular structure rather than a dumbbell-like shape. It is conceivable that the two ABDs are packed together in an approximately antiparallel arrangement with the N- and C-terminal CH domains (CH1 and CH4) making direct contact as shown in Figure 2(c); that is, *X*, *Y*, and *Z* correspond to CH1–CH3, CH2–CH4, and CH3-CH4 of the plastin protein, respectively. *P* indicates a putative N-terminal headpiece. See more details in [6, 7]. (c) Possible arrangement of the ABDs (CH1–CH4) of L-plastin in solution without Ca^2+^.

The structure of plastins is well conserved from lower eukaryotes to humans and is characterized by actin binding domains (ABD). An ABD consists of a pair of ~125 residue calponin-homology (CH) domains (Figure 2(a)). ABD-containing proteins include proteins such as spectrin, *α*-actinin, dystrophin, cortexillin, and plastin/fimbrin [2]. The plastins are unique among these, as they possess two tandem repeats of ABD (ABD1 and ABD2) within a single polypeptide chain and cross-link actin filaments into higher order assemblies like bundles through this tandem pair of ABDs [3]. N-terminal EF-hand Ca^2+^-binding domains (Figure 2(a)) are also important, since the actin bundling activity of plastins is regulated by Ca^2+^ [4].

Though ABD1 was previously solved by X-ray crystallography [5], the complete crystal structure of plastins has not yet been resolved. We have recently performed conformational analyses of murine L-plastin by X-ray scattering in solution and for the first time shown the overall structure of full-length plastin protein by reconstructing from data using the DAMMIN program (Figure 2(b)) [6]. The program DAMMIN is an advanced modeling procedure designed to reconstruct the shape of a molecule from the scattering intensity of small angle X-ray scattering data. Our results, taken together with those by Klein et al. on the plastin core region [7], demonstrated that plastin has a compact globular structure rather than a dumbbell-like shape and that the two ABDs are packed together in an approximately antiparallel arrangement with the N- and C-terminal CH domains (CH1 and CH4) making direct contact (Figure 2(c)). We also demonstrated that significant conformational changes of the protein were induced in the presence of Ca^2+^ [6]. These findings should shed light on the molecular mechanisms of how plastins regulate the architecture of actin filaments.

## 3. Functions of Plastin Isoforms

Since plastins are expressed in a cell-type-specific manner, it is conceivable that the most suitable isoform is expressed to regulate the actin cytoskeleton in a particular type of cell. Functions of plastin isoforms in distinct types of cells or tissues in mammals are reviewed.

### 3.1. Plastin Functions in Leukocytes

We isolated a 65-kDa cytosolic protein that was phosphorylated in murine macrophages by stimulation with bacterial lipopolysaccharide (LPS) and determined its complete primary structure as a novel protein [8–10]. The sequence of the 65-kDa protein revealed that it was a murine homolog of human L-plastin that had been identified as a transformation-induced polypeptide of neoplastic human fibroblasts [11]. We further demonstrated that L-plastin plays an important role in macrophage functions such as host defense against bacterial infections [12–15]. We, and others, have clarified that L-plastin is exclusively expressed in leukocytes such as lymphocytes, macrophages, and granulocytes under physiological conditions. Representative studies addressing the role of L-plastin in leukocyte functions are shown in Table 2 [8–10, 12–39]; the isoform serves important functions not only in cells of innate immunity such as macrophages and granulocytes (neutrophils and eosinophils) [8–10, 12–29], but also in those of adaptive immunity such as T and B lymphocytes [30–37].

 Many of the studies have addressed the phosphorylation of L-plastin during leukocyte activation by various stimuli (Table 2). Only L-plastin has, so far, been known to be phosphorylated in cells among three isoforms. We demonstrated that L-plastin was phosphorylated exclusively on Ser5 in macrophages by stimulation with LPS [10]. In connection with this, Jones et al. showed that the Ser5-phosphorylated L-plastin peptide induced adhesion in neutrophils [25]. It was also demonstrated that phosphorylation on Ser5 increased the F-actin-binding activity of L-plastin and promoted its targeting to sites of actin assembly in cells [40]. In addition to phosphorylation, the actin-bundling action of L-plastin is also regulated by intracellular Ca^2+^ [4]. The above findings indicate that L-plastin regulates the actin cytoskeleton in a phosphorylation- and/or Ca^2+^-dependent manner. These features of L-plastin seem to help leukocytes rapidly rearrange their actin cytoskeleton when they need to quickly respond to a variety of extracellular stimuli. Indeed, it has recently been demonstrated by using L-plastin gene-disrupted mice that neutrophils lacking L-plastin are deficient in killing bacterial pathogens [26] and that T and B cell functions are also impaired in the mice; T cell responses to antigens are impaired [36], and splenic maturation of B cells and an antibody response to *Streptococcus pneumoniae* are largely diminished [37].

 These studies regarding the key roles of L-plastin in leukocytes functions may provide the basis for new therapy. Several suppression immunotherapies targeting L-plastin have been proposed (Table 2) [38, 39]. Cannabinoid CB2 receptor-specific compounds were found to inhibit the L-plastin phosphorylation in human monocytes and blocked experimental autoimmune encephalomyelitis in the rat [38]. In addition, it has recently been shown that glucocorticoid dexamethasone inhibits the L-plastin phosphorylation in human T cells, which prevents the immune synapse formation and subsequent T cell activation [39].

### 3.2. Plastin Functions in Neurons

The nervous system chiefly expresses T-plastin among the three isoforms. Though microglia in the brain are a kind of resident macrophage, their expression of L-plastin is very low under physiological conditions (unpublished observations). With regard to T-plastin in neurons, an interesting article has recently been published; the authors showed that high expression of T-plastin acts as a protective modifier of spinal muscular atrophy (SMA), the most frequent genetic cause of early childhood lethality, and that T-plastin is important for axonogenesis, as its overexpression rescues the axon length and outgrowth defects in neurons of the SMA mouse [41]. Therefore, T-plastin seems to play a vital role in neuronal differentiation. Another interesting study addressed the preventive role of T-plastin in neurodegenerative diseases, demonstrating that T-plastin lessens the toxicity of polyglutamine proteins in neurons, such as ataxin and huntingtin, that cause neurodegenerative disorder [42]. These, and related studies, are summarized in Table 3 [41–43].

Since sensory cells are closely related to the nervous system, studies regarding the plastin expression in sensory cells are reviewed here as shown in Table 3. Several studies using specific antibodies against plastins/fimbrin revealed that the proteins are expressed in the stereocilia of auditory hair cells in the chicken and mammals [44–47], suggesting that plastins may contribute to the physiological auditory sense. In the rat cochlear hair cells, T- and I-plastin, but not L-plastin, were found to be expressed [47]. T-plastin is expressed only in the early stage of hair cell differentiation, while I-plastin is constantly expressed from the early to the adult stages, indicating that I-plastin is the major isoform expressed in the adult cochlear hair cells. The architecture of actin filaments cross-linked by plastins may be essential for the transduction of auditory signals by cochlear hair cells.

### 3.3. Plastin Functions in the Intestine

The first protein of the plastin/fimbrin family was discovered in microvilli of the chicken intestinal brush border and named fimbrin [48]. Chicken fimbrin was characterized as a cytoskeletal protein that binds and cross-links F-actin filaments [49, 50]. Meanwhile, a third plastin isoform, named I-plastin, was identified in humans and was found to be expressed in the small intestine, colon, and kidney [51]. Thus, I-plastin is considered to be the human homolog of chicken fimbrin. Using I-plastin gene-disrupted mice, it has recently been reported that a lack of I-plastin results in increased fragility of the intestinal epithelium and decreased transepithelial resistance [52], suggesting that I-plastin is an important regulator of brush border morphology and stability. Recently, a model of the microvillar cytoskeleton of the brush border that includes plastin/fimbrin cross-linking actin filaments has been proposed [53]. These studies are summarized in Table 4 [48–53].

### 3.4. Plastin Functions in Cancer

The cell-type-specific expression of plastin isoforms is strictly regulated under physiological conditions. However, ectopic expression of plastins in malignant cells has been observed in many studies as shown in Table 5 [11, 54–69]. L-plastin that is normally expressed only in hematopoietic cells is especially expressed in a variety of cancer cells of nonhematopoietic origin. Lin et al. demonstrated that 68% of cancers derived from epithelia express L-plastin [54]. It was further clarified by using sensitive RT-PCR that the L-plastin gene is activated in most human cancer cells [56]. Thus, L-plastin has been considered to be a common marker of many types of human cancer. In particular, a high percentage of cancer cells arising from female reproductive tissues express L-plastin constitutively and abundantly though its expression is ovarian steroid hormone-independent in cancer cells [58]. In addition, the expression of L-plastin in prostatic epithelial cells is linked to the malignant state, and once expressed in carcinoma, its expression is regulated by steroid hormone receptors [59]. The L-plastin gene promoter was found to include several hormone receptor-responsive elements [55, 57]. These evidences support a *trans*-activation mechanism for the activation of L-plastin synthesis accompanying tumorigenesis. In contrast, T-plastin gene expression was found to be suppressed in human colorectal cancer cells, suggesting that downregulation of T-plastin is involved in cancer development [65].

 Since L-plastin is normally expressed in leukocytes that are able to move rapidly to infectious and inflammatory sites, cancer cells may gain the ability to metastasize to other parts of the body by expressing L-plastin. In other words, L-plastin tends to be expressed in freely movable cells such as leukocytes and cancer cells. On the other hand, it was found that T-plastin is expressed in certain types of malignant cells of leukocyte origin, including cutaneous T cell lymphoma and Sezary syndrome (Table 5) [66–69]. In cutaneous T cell lymphoma, dense clusters or nodules of malignant cells are observed [70]; these cells appear to lose their motility. This seems to contrast sharply with the case of cancer cells that express L-plastin. Considering the above, the expression of plastin isoforms could be dysregulated when cells, regardless of their origin, become malignant, which may endow tumor cells with properties distinct from those of their normal counterparts.

 On the basis of these observations regarding abnormal expression of plastins, clinical applications have been developed. Cancer screening and diagnosis methods assessing the expression of plastins as a biomarker have been described. These include choroids plexus tumors, urinary bladder cancer, ovarian cancer, and colorectal cancer (Table 5) [71–74]. Furthermore, gene therapy experiments targeting the L-plastin gene or L-plastin promoters in cancer cells have been started, as shown in Table 5 [75–78]. These approaches have shown promising results. For example, Peng et al. prepared adenoviral vectors in which a truncated human L-plastin promoter and the *cytosine deaminase* (*CD*) gene were coinserted [76]. *CD* is a bacterial gene which converts 5-fluorocytosine (5FC: nontoxic to cells) to 5-fluorouracil (5FU: toxic to most cells). When the vector is transfected into human ovarian or bladder cancer cells *in vitro*, CD transcription is increased through the L-plastin promoter activation, leading to tumor cell death via the conversion of 5FC to 5FU in the cells. The authors also performed *in vivo* study and demonstrated that human tumor masses grown in nude mice were reduced by this method [76]. In another study by Zheng et al., the authors constructed retroviral vectors to express regions of the human L-plastin gene in antisense orientation and found that introduction of the vectors into prostate carcinoma cells reduced the growth rates of the cells and suppressed their invasion and motility *in vitro* [78]. This suggests that overexpression of L-plastin is involved in cancer invasion and metastasis and that downregulation of L-plastin by antisense delivery is potentially a useful approach to interfere with prostate cancer progression.

## 4. Perspectives

Since plastins are evolutionarily conserved and expressed in yeast, plant, and animal cells, they should play a fundamental role in cellular activities. Sophisticated regulation of the actin cytoskeleton seems to be a mandatory event for the functions of eukaryotic cells. It has been investigated in detail how plastins interact with actin filaments *in vitro*. Extensive studies have clarified that three plastin isoforms are expressed in distinct types of cells in mammals and that dysregulated expression of plastins appears to be important in the progression of various cancers. As described in this paper, plastins can serve as versatile regulators of the actin cytoskeleton in many aspects of cellular functions. Further studies will be expected to reveal how plastins, together with other actin-binding proteins, dynamically regulate the remodeling of cytoskeletal architecture during diverse cellular activities.

## Figures and Tables

**Table 1:** Plastin isoforms expressed in distinct cell types.

Isoforms	Cell/tissue types
I-plastin (Plastin 1)	Intestine and kidney
L-plastin (Plastin 2)	Leukocytes and cancer
T-plastin (Plastin 3)	Solid tissues

**Table 2:** Studies on L-plastin in leukocytes.

Leukocyte types	Key words of the study	References
*Innate immunity*		
Macrophages	Bacterial lipopolysaccharide (LPS)-induced activation	[8–10, 12]*, [13, 14]
Macrophages	Grancalcin and host defence	[15]
Macrophages	IL-1/TNF-induced activation	[16]*
Macrophages	Podosome formation	[17]
Macrophages	Zebrafish and lineage marker	[18–20]
Macrophages	*Toxoplasma gondii*-infection	[21]
Neutrophils	IL-8-induced activation	[22]*
Neutrophils	Fc receptor and phagocytosis	[23, 24]*
Neutrophils	L-plastin KO^#^ and integrin	[25, 26]*
Eosinophils	GM-CSF-induced priming	[27]*
Osteoclasts	Podosome formation	[28]
Osteoclasts	Sealing ring formation	[29]
*Adaptive immunity*		
T-cells	IL-2-induced activation	[30]*
T-cells	Accessory receptors	[31]*
T-cells	Lymphokine-activated killer cells	[32]*
T-cells	Costimulation	[33]*
T-cells	CCR7 and thymus	[34]
T-cells	LFA-1 and immune synapse	[35]
T-cells	L-plastin KO^#^ and impaired T cell responses	[36]
B-cells	Marginal zone B cell development	[37]
*Suppression immunotherapies targeting L-plastin*		
Leukocytes	Cannabinoid receptor agonists	[38]
Leukocytes	Glucocorticoid dexamethasone	[39]

*These studies addressed the L-plastin phosphorylation in leukocytes.

^#^Disruption of the L-plastin gene in mice.

**Table 3:** Studies on plastins in the nervous system and sensory cells.

Key words of the study	References
*T-plastin in neurons*	
Spinal muscular atrophy and axonogenesis	[41, 43]
Spinocerebellar ataxia and polyglutamine protein	[42]
*Plastin/fimbrin in sensory cells*	
Fimbrin, chicken, stereocilia, auditory hair cells	[44, 45]
I-plastin, mouse/rat, auditory hair cells	[46, 47]

**Table 4:** Studies on fimbrin/I-plastin in the intestine.

Key words of the study	References
Fimbrin, Microvilli	[48]
Intestinal brush border	[49, 50]
I-plastin, Intestine, Kidney	[51]
I-plastin KO^#^, Intestinal epithelium	[52]
Model of microvillar cytoskeleton	[53]

^#^Disruption of the I-plastin gene in mice.

**Table 5:** Studies on plastins in cancer cells.

Key words of the study	References
*Ectopic expression of L-plastin in cancer*	
Transformed human fibroblasts	[11]
Many types of human cancer	[54]
L-plastin gene promoter in cancer	[55–57]
Ovarian steroid hormones	[58]
Prostate cancer and steroid hormone	[59]
Chromosome translocation	[60]
Breast cancer and expression pattern	[61]
Colorectal cancer and metastasis	[62]
Colon cancer, invasion, and loss of E-cadherin	[63]
Melanoma tumor invasion	[64]
T-plastin downregulation and CpG methylation	[65]
*Ectopic expression of T-plastin in lymphoma*	
Cutaneous T cell lymphoma	[66, 67]
Sezary cells	[68, 69]
*As a biomarker for cancer screening and diagnosis*	
Choroid plexus tumors and diagnostic marker	[71]
Bladder cancer and biomarker	[72]
Proteomics imaging and mass spectrometry	[73]
Colorectal cancer and human feces	[74]
*Gene therapies targeting L-plastin gene*	
L-plastin promoter and gene therapy	[75–77]
Antisense L-plastin gene and tumor suppression	[78]

## Abbreviations

ABD: Actin binding domain
CH: Calponin-homology
IL: Interleukin
LPS: Bacterial lipopolysaccharide
SMA: Spinal muscular atrophy
TNF: Tumor necrosis factor.
